# Imaging striae distensae: a comparison between PS-OCT and digital dermoscopy

**DOI:** 10.1364/BOE.417084

**Published:** 2021-05-11

**Authors:** Wai Ching Lin, Robert A. Byers, Wei Li, Simon G. Danby, Michael J. Cork, Stephen J. Matcher

**Affiliations:** 1 Department of Electronic and Electrical Engineering, University of Sheffield, Sir Frederick Mappin Building, Sheffield, S1 3JD, UK; 2 Sheffield Dermatology Research, Department of Infection and Immunity and Cardiovascular Disease, University of Sheffield, Beech Hill Road, Sheffield, S10 2RX, UK

## Abstract

Stretch marks or striae distensae (SD) cause emotional distress and negatively affect the psychological well-being of patients. We investigate and compare two methods for quantifying the severity of SD: visual scoring of images captured using a clinical visible-light dermatological camera (C-Cube, Pixience Inc) and measuring the local birefringence of skin using polarization-sensitive optical coherence tomography (PS-OCT). Data on skin visually affected by SD and visually normal skin were collected from 19 human volunteers. Our results show a weak correlation between visual scores of the C-Cube images and the birefringence values obtained from the PS-OCT system. SD datasets have a significantly larger birefringence values compared to visually normal datasets.

## 1. Introduction

Striae distensae (SD), more commonly known as stretch marks, are dermal scarring lesions on the skin [1]. They are caused by excess mechanical stretching of the dermis over time [2, 3], an increase in hormonal receptor activity [4] and/or a reduced expression of collagen, elastin and fibronectin [5]. An initial reaction in dermal collagen expression is hypothesized to mechanically weaken the dermis and make it susceptible to tearing. The resulting wound-healing response lays down scar tissue of highly aligned collagen and elastin, which forms the visible SD. They are aesthetically undesirable, place a psychosocial burden on patients and negatively affect their quality of life [6,7]. SD often occurs as a result of pregnancy (striae gravidarum), obesity and growth spurts [8]. Risk factors for SD include Cushing syndrome and chronic corticosteroid usage [3]. Treatment for SD typically involve the use of topical products, either therapeutically or prophylactically [1]. Topical tretinoin is one such product [9,10]. Chemical peel treatments [11], Galvano-puncture [12] and laser-based treatments [13] are also available. The goal of most treatments is to stimulate collagen production and realignment [6].

SD typically begin as reddish lesions [1] called striae rubra (SR). These appear as raised, erythematous regions with a pink to reddish coloration [8]. Over time, the epidermis gradually atrophies and SR progresses into atrophic, hypopigmented (white) lesions called striae alba (SA). SA lesions are flatter and more depressed compared to surrounding healthy tissue [14].

Currently, no gold standard exists for quantifying SD severity. Many studies and clinical trials investigating treatment efficacy used subjective scoring systems that vary from study to study [6]. Some studies numerically rate photographs of SD skin [9] or survey patient satisfaction [10], while others measure the length and width of SD lesions [10]. Biopsies for histological staining are also commonly used for a more objective but still qualitative measurement [9,10]. A recent study by Cho et al. [8] compared SD skin and healthy skin by measuring a number of biophysical properties. They found that SD skin had higher surface roughness [8,14], lower skin elasticity [8] and lower dermal echo density [8]. However, the study did not attempt to quantify SD severity through these methods. There is a need for objective measurement tools that can quickly and accurately detect the localized health of the skin. The reliance of such a tool on subjective evaluation should be minimal. To develop such a measurement standard, it is necessary to have a marker that can quantify a physiological property of the skin that differentiates SD skin from normal skin.

Optical coherence tomography (OCT) is a non-invasive imaging modality that detects near-infrared light backscattered from the tissue sample. It produces high-resolution cross-sectional images of tissue morphology. It has a penetration depth of a few millimeters [15] and is capable of imaging the epidermis and dermis of skin tissue [16]. Polarization-sensitive (PS) OCT is a functional extension to OCT. Polarimetric information measured by the PS-OCT is used to find the phase retardance [17,18] and fast birefringent axis orientation [19] of the sample. The phase retardance is especially useful in estimating birefringence, the degree of fiber alignment in tissues. This is particularly useful in fibrous tissues with a high degree of collagen and elastin. PS-OCT has been successfully utilized in dermatology [16], ophthalmology [20], dentistry [21] and an enhanced study of bones, tendons, ligaments and cartilage [22]. To date, there have been no known studies measuring the birefringent properties of SD skin.

The onset of SD results in changes to the collagen and elastin fiber alignment within the skin. In healthy skin, elastin and collagen fibres are arranged in a random orientation [6]. Regardless of the specific cause, SD onset results in an over-production of elastin and collagen over time. Histological specimens show densely packed elastin and collagen fibres aligned parallel to the skin [6]. We hypothesize that this increase in fibre alignment will result in an increase in the localized phase retardance, leading to quantifiable differences in the birefringence between SD and healthy skin.

This paper aims to assess the feasibility of using PS-OCT in objectively quantifying SD severity. It is an exploratory study to measure the birefringence of SD and healthy skin. Results from the PS-OCT measurements will be compared against subjective visual scoring.

## 2. Method

### 2.1 Participants

This study was favourably reviewed by The University of Sheffield Research Ethics Committee (UREC #030680). 19 adult volunteers were recruited and the striae in different locations around their body were imaged. Participants were screened against other skin conditions such as eczema, acne, suntan, hyperpigmentation, tattoos, blemishes and dense body hair.

A total of 75 datasets containing striae were collected. These were visually inspected at the time of screening to categorize them as either striae rubra or striae alba. For each dataset, a corresponding dataset at a site adjacent to the affected skin was also collected. This adjacent dataset is hereafter referred to as “healthy” data, whilst recognizing that it may possibly correspond to sub-clinical SD skin.

### 2.2 C-Cube imaging and scoring

A clinical visible-light dermatological camera (C-Cube, Pixience Inc, France) was used to capture high-resolution close-up en-face images of the skin. It is able to image the region of interest under controlled lighting conditions, thus giving a more objective view of the striae lesions that is unaffected by surrounding ambient light intensity and direction. The camera also has a mode that estimates surface profiles from shading, but this mode was not used here. If the participant presents with more than one striae lesion, multiple scans were acquired. Each 2D C-Cube image covers an area of approximately 16×12 mm. A black marker, approved for dermatological use, was used to mark the center of the images to ensure the same area was imaged by both C-Cube and PS-OCT. Visual scoring of the striae-affect C-Cube images was done using the criteria shown in Table 1. The images were scored by 3 individuals independently and then the arithmetic mean of these scores was reported. To our knowledge, there is no agreed upon scoring system for quantifying SD severity. In a comparable study, Rangel et al have used a scoring system for quantifying SD severity by rating photographs [9]. However, this scoring system gave a numerical score based on the improvement in SD lesions after treatment rather than the severity of each lesion. Therefore, the scoring system used here was generated in our lab for this study.

**Table 1.** Criteria for visual scoring of striae using C-Cube images

Score	Description of c-cube image
**0**	**No visible striae**
**1**	**Barely perceptible striae**
**2**	**Visible striae**
**3**	**Clear visible striae with evidence of deep furrows**

### 2.3 PS-OCT data collection

**Fig. 1.**
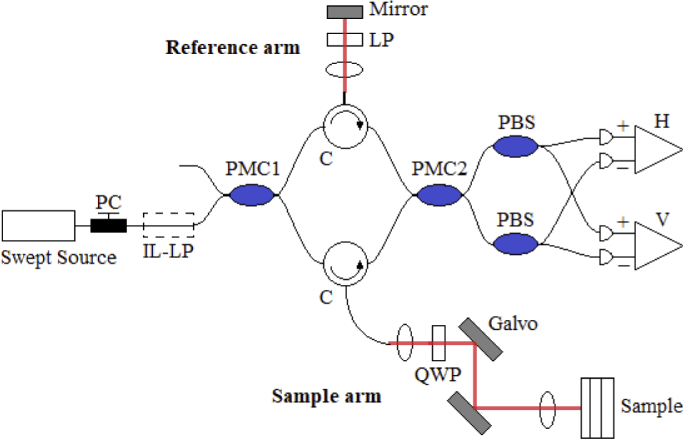
Schematic diagram of PS-OCT system. A swept-source light beam passes through a polarization controller (PC) and an in-line polarizer (IL-LP). A polarization-maintaining coupler (PMC1) and PM circulators (C) split the beam into reference and sample arms in a 10:90 ratio. In the reference arm, the beam passes through a linear polarizer (LP) and reflects off a mirror. Light in the sample arm is passed through a quarter wave plate (QWP) before reaching the sample. 2 galvanometers (Galvo) allow volumetric scanning. Interference from both arms occur at a second PM coupler (PMC2). The signals are separated using 2 polarizing beamsplitters (PBS) into horizontal (H) and vertical (V) channels before being detected by 2 balanced detectors.

The OCT system used is a polarization-maintaining (PM) fiber-based PS-OCT system. Its configuration was first reported by Al-Quasi et al in [23]. A more detailed report of our in-house system can be found in [24]. Figure 1 shows a schematic diagram of the setup. The measured axial resolution of the system is approximately 10 µm in air. The light source used is a commercially available swept-source laser (HSL-2000-10-MDL, Santec, Japan) with a center wavelength of 1315 nm and a full-width-half-maximum (FWHM) of 128 nm. It has a sweep range of 157 nm and a sweep rate of 10 kHz. The laser has a duty cycle of 60% and an output power of 10mW.

The setup in Fig. 1 is a Mach-Zehnder interferometer that uses Panda PM-fibers. The laser output beam first passes through a polarization controller (PC) and an in-line linear polarizer (IL-LP), which ensures linearly polarized light. Using a 2 × 2 PM coupler (PMC1, OLCPLP-22-131-10-90-FA, Opto-link Corp., China), the light beam is split into the reference arm and sample arm in a 10:90 ratio. A pair of three-port PM circulators (C, OLCIR-P-3-131-300-90-FA, Opto-link Corp., China) direct the light beams to the sample and reference reflector. In the reference arm, the light beam passes through a linear polarizer (LP) oriented at 45° to the slow axis of the PM-fiber before being reflected by a static plane mirror. Light in the sample arm is passed through a quarter wave plate (QWP, NT55-547, Edmund Optics, USA) whose fast-axis is oriented at 45° to the fast-axis of the PM fiber, thus converting the polarization to a circular state before reaching the sample. A set of 2 galvanometers (Galvo, 6215, Cambridge Technology, USA) raster scan the beam in the en-face plane. Light from both arms interfere when they reach the second PM coupler (PMC2, OLCPL-P-22-131-50-90-FA, Opto-link Corp., China). After the light beam is separated into horizontal (H) and vertical (V) polarization states using polarizing beam splitters (PBS, PBS-31-P-2-L-3-Q, NovaWave Techno., USA), they are detected by two balanced detection channels (1817-FC, New Focus, USA). A 14-bit transient recorder (M2i.4022, Spectrum GmbH, Germany) is used to measure the two signals at a 20 MS/s sampling rate.

A MATLAB graphical user interface (GUI) was used to control the system and capture volumetric C-scans.

### 2.4 PS-OCT image processing

Each volume scan covers an area of 6×6mm with a lateral step size of 10 µm. All data processing steps were done in MATLAB (2019a, MATLAB) with the Signal Processing, Image Processing and Wavelet toolboxes. A fast Fourier transform (FFT) was performed on each raw interferometric signal to obtain the intensity profile of each A-scan. A wavelet-FFT filtering algorithm was applied to remove the presence of ghost artifacts in the B-scans, which are caused by mode dispersion in the PM fibers [25]. A surface flattening algorithm was also used to detect and flatten the surface of each C-scan [25].

Both the amplitude and phase of the horizontal and vertical signals were used to calculate the Stokes vector (*S*) of each voxel. The Stokes vector was computed using Eq. (1), which was taken from [26].

(1) $$S=\left(\begin{array}{c} I\\ Q\\ U\\ V \end{array}\right)=\left(\begin{array}{c} {A_{V}}^{2}+{A_{H}}^{2}\\ {A_{V}}^{2}-{A_{H}}^{2}\\ 2A_{V}A_{H}\cos\Delta \phi \\ 2A_{V}A_{H}\sin\Delta \phi \end{array}\right)$$

*I*, *Q*, *U* and *V* are elements of the Stokes vector. $A_{H,V}$ are the amplitudes of the horizontal and vertical channels respectively, while Δϕ is the phase difference between the vertical and horizontal signals. The Stokes vector in Eq. (1) is further normalized by dividing the *Q*, *U*, *V* values by *I*, as shown by Eq. (2). The conversion of Stokes vectors into an enface image of birefringence broadly follows the procedure described by Chin et al. [27].

(2) $$\hat{S}=\left(\begin{array}{c} \hat{Q}\\ \hat{U}\\ \hat{V} \end{array}\right)=\left(\begin{array}{c} \text{Q}/\text{I}\\ \text{U}/\text{I}\\ \text{V}/\text{I} \end{array}\right)$$

The phase retardance at a discrete depth *z_i_* ($\phi_{r}$) can be calculated by taking the dot product between the Stokes vector at the surface ${\hat{S}}_{ref}$and the Stokes vector at *z_i_* [Eq. (3)]. The derivation of Eq. (3) is explained in greater detail by Chin et al. [27]. On the assumption of weakly varying sample fast-axis orientation with depth, local birefringence can then be estimated by the local gradient of $\phi_{r}$ with respect to depth. To give a more precise estimate of the gradient and reduce the effects of random noise, 2D averaging using a 3×3 pool is performed on the Stokes vectors before the dot product is computed. In other words, the averaged Stokes vector was calculated by finding the arithmetic mean of every 3×3 block of Stokes vectors.

(3) $$cos\;\phi_{r}(z_{i})={\hat{S}}_{ref}\cdot \hat{S}(z_{i})$$

The phase retardance profile $\phi_{r}(z_{i})$ is demodulated by forming the analytic signal of Eq. (3) and finding the resulting phase angle, which yields the demodulated wrapped phase retardance $\phi_{w}$. The latter is unwrapped by using the *unwrap* function in MATLAB. Using a simple linear regression, the slope of the unwrapped phase retardance was calculated at a depth of 3–53 pixels from the surface of the skin, which correlates to a depth of 12–212 µm. The slope of the unwrapped phase retardance $\delta \phi_{w}$ is converted to an estimate of the tissue birefringenceΔn using Eq. (4):

(4) $$\Delta n=\frac{\delta \phi_{w}(\lambda_{0})(RI)}{4\pi (\Delta z)}$$

where $\lambda_{0}$ is the central wavelength of the PS-OCT system (1301 nm), Δz represents the pixel size in air (4 µm) and RI is the refractive index of tissue (1.4). This estimate of the tissue birefringence *Δn*, calculated using the slope of phase retardance, represents the combined effect of local birefringence and fast optic axis of the tissue.

An important caveat about our measurements of tissue birefringence is that we measure what we have previously termed the “apparent birefringence”. This only equals the “true birefringence” when the collagen fibers are aligned parallel to the skin surface [24]. The clinical etiology of SD is that they orient themselves along tears in the dermal collagen, i.e. along directions of maximum tensile stress [28]. Therefore, we consider it reasonable to assume that the fast optic axis is constant with respect to depth and the slope of phase retardance is proportional to tissue birefringence. As healthy skin consists of randomly oriented collagen and elastin fibres, the slope of the phase retardance in healthy skin is expected to be approximately zero.

### 2.5 Thresholding and (un)wrapping

**Fig. 2.**
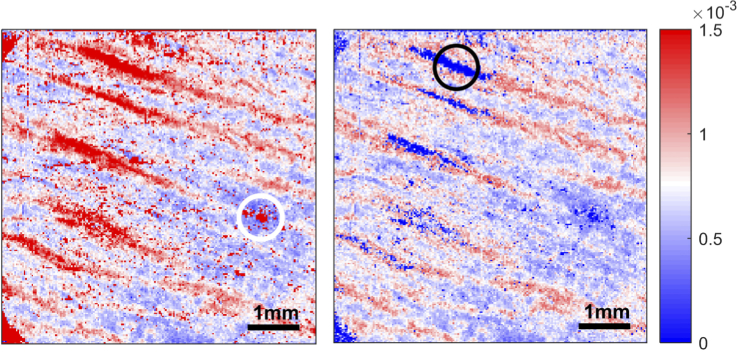
Example of tissue birefringence image with artifacts. The overall C-Cube visual score is 1. Left: Local tissue birefringence estimated using the unwrapped phase retardance. Areas with striae show high tissue birefringence, but areas where there should be no striae (white circle) show uncommonly high tissue birefringence. Right: Local tissue birefringence estimated using wrapped phase retardance. Areas where striae are located (black circle) should have high tissue birefringence (>1.5×10^−3^) but instead the tissue birefringence calculated is close to zero.

Unwrapping the phase retardance prior to slope estimation produced artifacts within the tissue birefringence image in areas of low expected tissue birefringence. In Fig. 2 (left), the en-face tissue birefringence image was calculated with the phase retardance $\phi_{w}$ unwrapped. Areas with striae show high tissue birefringence (>1.5×10^−3^) as expected, but areas where there should be no striae, for example in the white circle, also seem to have high tissue birefringence. Conversely, when the tissue birefringence image was calculated using the wrapped phase retardance, as in Fig. 2 (right), areas without striae have low tissue birefringence. However, areas of high tissue birefringence, for example in the black circle, also have very low (close to zero) tissue birefringence. To understand the reason for this, we look at the phase retardance profiles at these locations.

**Fig. 3.**
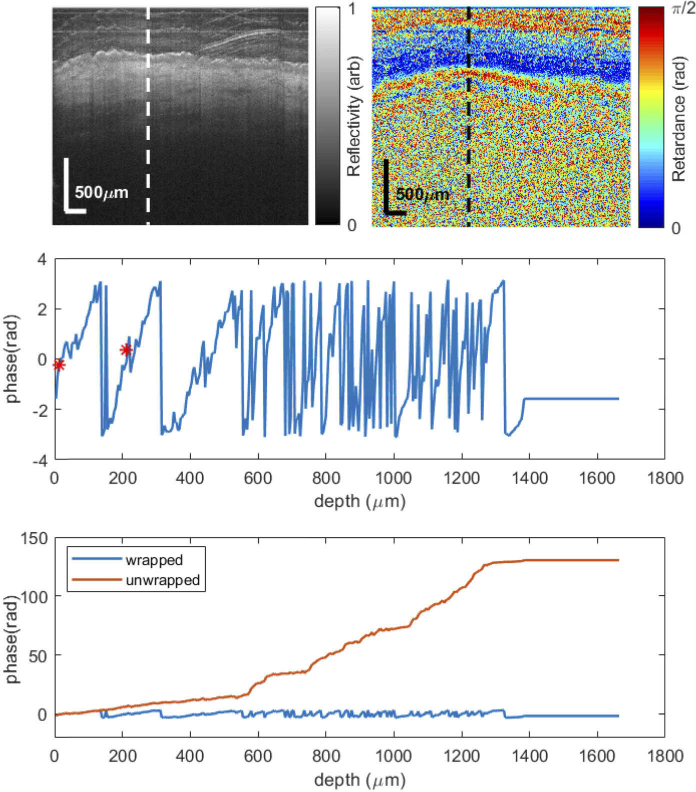
Demodulated wrapped and unwrapped phase retardance ($\phi_{w}$) profile of a region with striae (high expected birefringence). Top: Structural and retardance images of the region of interest. The dashed vertical line shows the shows the A-scan location used for the phase retardance profiles. Centre: close-up of the wrapped phase retardance. The phase increases as the depth increases. At 136 µm, the phase ‘wraps’ and decreases sharply by 2π. The slope is estimated using the profile between the two red points. Bottom: graph of both the wrapped and unwrapped phase. When the phase is unwrapped, slope estimation between the 2 points is positive and indicative of the true birefringence value. After 600 µm, noise becomes more prevalent and the unwrapping algorithm is unable to reliably detect a true phase wrap from noise.

Firstly, Fig. 3 shows the demodulated wrapped phase retardance ($\phi_{w}$) profile of an A-scan in the black circle in Fig. 2. The structural and retardance images at the top of the figure include a dashed vertical line, which shows the A-scan location used to calculated the phase retardance profile. The centre graph shows a close-up of the wrapped phase retardance with respect to depth. The phase increases as the depth increases. At a depth of 136 µm, the phase ‘wraps’ and decreases sharply by 2π. However, the slope of the phase profile before and after this phase jump is similar. With increasing depth, noise becomes more prevalent and it is impossible to reliably determine where other phase wraps occur. Slope estimation by linear regression occurs in between the 2 red dots at a depth of 12-212 µm. The slope calculated is close to zero due to the phase wrap.

The bottom graph in Fig. 3 includes both the wrapped and unwrapped phase profile. When the phase is unwrapped, slope estimation between the 2 points is positive and an accurate indication the birefringence. After 600 µm, where noise becomes more prevalent, the unwrapping algorithm is unable to reliably determine a true phase wrap. Noise fluctuations cause the unwrapping algorithm to incorrectly add 2π phase offsets at numerous locations. Thus, the slope after 600 µm becomes artifactually higher than the slope calculated at 12–212 µm.

**Fig. 4.**
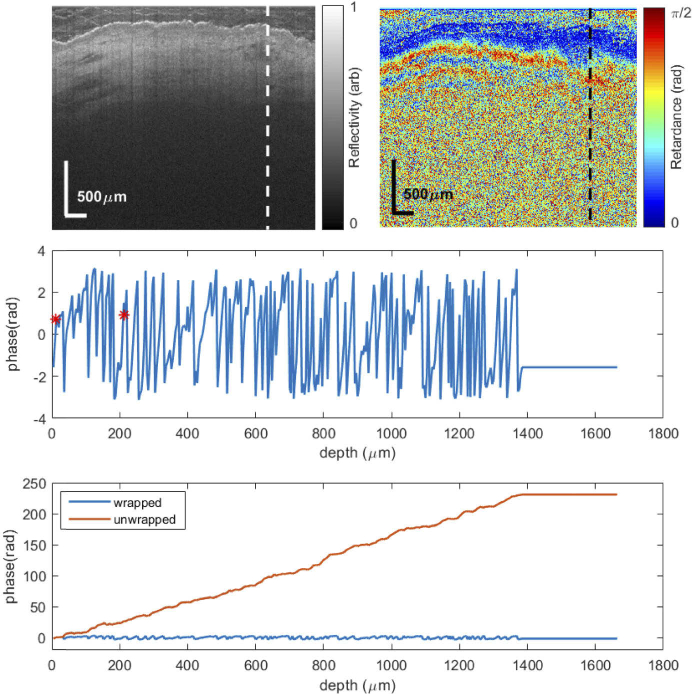
Demodulated wrapped and unwrapped phase retardance profile of a healthy region (low expected birefringence). Top: Structural and retardance images of the region of interest. The dashed vertical line shows the shows the A-scan location used for the phase retardance profiles. Centre: close-up of the wrapped phase retardance profile. There are no clear signs that a wrap has occurred within this phase profile. The slope estimated between the two red points is approximately zero. Bottom: wrapped and unwrapped phase profiles. At a depth of 12–212 µm, the phase increases from 0 to 27 rad. At the same depth in Fig. 3, the phase only increased by 6 rad. Most of the unwrapped phase jumps in this graph is due to noise.

Figure 4 shows a phase retardance profile of a voxel within the white circle in Fig. 2, a low-birefringence (based on visual inspection) region of a striae image. This is a region without striae and tissue birefringence is expected to be close to zero. There are no clear signs of a true phase wrap within this phase profile. In fact, the entire phase profile is dominated by noise. The slope estimated is thus approximately zero. The bottom graph in Fig. 4 shows both the wrapped and unwrapped phase. When the phase is unwrapped, it increases at a higher rate compared to the unwrapped phase in Fig. 3. For instance, in Fig. 4, at a depth of 12–212 µm, the phase increases from around 0 to 27 rad. At the same depth, the phase in Fig. 3 only increased by 6 rad. This is because MATLAB’s *unwrap* function cannot distinguish between a true phase wrap and noise. Most of the unwrapping in Fig. 4 is due to noise, and the subsequent slope and hence tissue birefringence calculated are not indicative of striae severity. In these instances of high noise and low tissue birefringence, the slope estimated using the wrapped phase is more accurate.

**Fig. 5.**
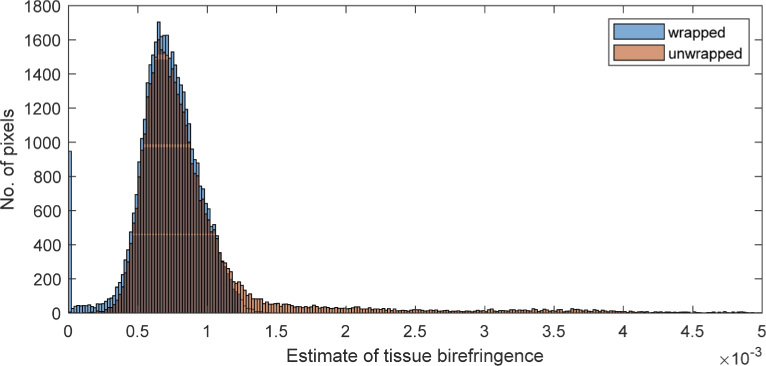
Histogram of wrapped and unwrapped enface local tissue birefringence image. In the wrapped histogram, there are a large number of pixels with zero tissue birefringence. The maximum tissue birefringence calculated in the wrapped image is around 1.5×10^−3^. The unwrapped histogram has a greater range of tissue birefringence values, but the histogram is truncated at 5×10^−3^ for ease of viewing.

**Fig. 6.**
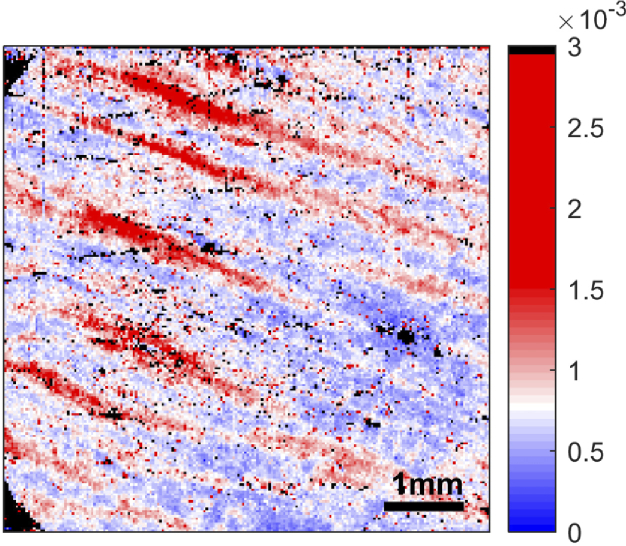
Enface image of tissue birefringence (estimated using the unwrapped phase), with black pixels highlighting areas where the tissue birefringence is larger than 3×10^−3^.

To improve the accuracy of tissue birefringence estimations, it is necessary to separate voxels of high noise from those with genuinely high tissue birefringence. Figure 5 shows histograms of the wrapped and unwrapped enface images. For the wrapped image, there are a large number of pixels with zero tissue birefringence due to the aforementioned issues. The zero-slope estimate means the voxel is either a non-striae region or a striae region with a wrapped phase. The maximum tissue birefringence calculated in the wrapped image is around 1.5×10^−3^. The unwrapped image had a greater range of tissue birefringence values. There are a small number of pixels with tissue birefringence larger than the 5×10^−3^ shown in the graph. They have been truncated from this histogram for ease of viewing. As discussed above, regions of low tissue birefringence and high noise tend to have slopes (and hence estimated tissue birefringence) that are higher than striae regions, when unwrapped phase profiles are used. Tissue birefringence values are first estimated using the unwrapped phase. By looking at the histogram plot, we can select a threshold (for this dataset, it is 3×10^−3^). For any pixel exceeding this threshold, the tissue birefringence value is re-calculated using the original, wrapped phase profile. Figure 6 highlights all pixels above this threshold in black. Only healthy regions with low expected tissue birefringence are highlighted.

**Fig. 7.**
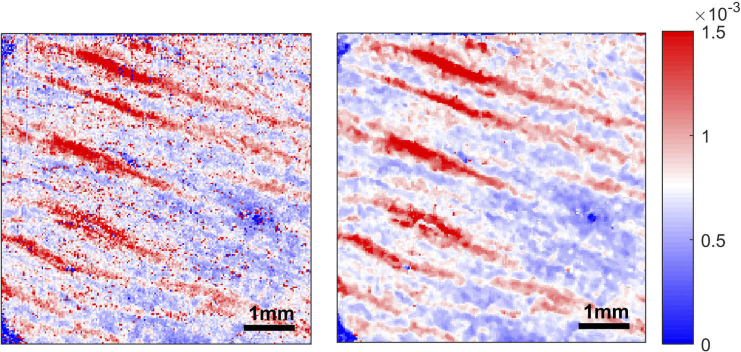
Enface image of tissue birefringence after thresholding. Left: Pixels with a tissue birefringence value higher than 3×10^−3^ use the wrapped phase for slope estimations, and vice versa. Right: Enface image of tissue birefringence after thresholding and applying a 3×3 median filter.

Figure 7 (left) shows the result of using this thresholding. Areas with striae now have high tissue birefringence values (up to the threshold of 3×10^−3^), while areas without striae have low tissue birefringence values.

However, speckle noise is still present in the image, which could affect the statistical analysis of tissue birefringence values within the image. To reduce this noise, a 3×3 median filter is applied. Figure 7 (right) shows the enface image after median filtering. While the image resolution is reduced, the speckle noise has also reduced.

Thresholding was only applied to images where it is clear that the areas without striae have high birefringence. The threshold is selected individually for each image. If the threshold chosen is too high, areas without striae would still have a high tissue birefringence. The modified image would look like the image in Fig. 2 (left). If the threshold is too low, regions of striae in the modified image would have low tissue birefringence. The resultant image would look similar to Fig. 2 (right).

## 3. Results and discussion

**Fig. 8.**
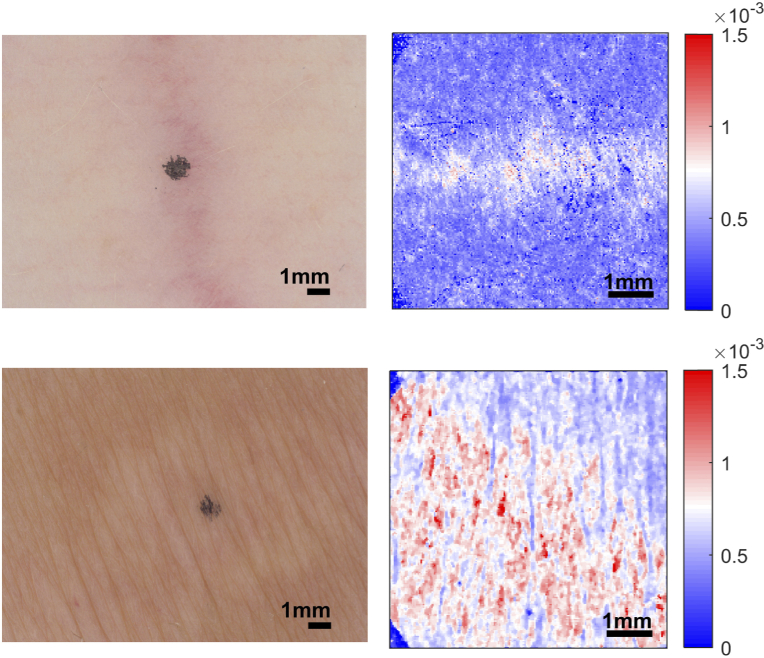
Examples of striae rubra (top) and striae alba (bottom). The images on the left are from the C-Cube camera; the images on the right from the PS-OCT. Striae rubra: overall visual score of 1. Striae alba: overall visual score of 2. N.B. the angular offset is not matched between the different imaging modalities.

A total of 150 datasets were collected, 75 samples with striae and 75 samples of adjacent healthy skin. Each dataset comprises of a photograph captured by the C-Cube camera and a volume scan measured using the PS-OCT system. Of the samples with striae, 60 were of striae alba, 11 were of striae rubra and the remaining 4 were classified as a mixture of both. Figure 8 shows an example of striae rubra and striae alba. The images on the left were taken using the C-Cube camera and the images on the right show the enface images of tissue birefringence. N.B. the angular offset is not matched between the different imaging modalities. The top image in Fig. 8 is an example of striae rubra, which has an overall visual score of 1. The bottom image, an example of striae alba, has a score of 2. In the tissue birefringence images, there are clear differences between areas of healthy skin (low tissue birefringence, coloured blue) and areas of striae (high tissue birefringence, coloured red). Moreover, the sample with striae alba is visibly more severe than the sample with striae rubra in terms of the area covered and the level of tissue birefringence.

**Fig. 9.**
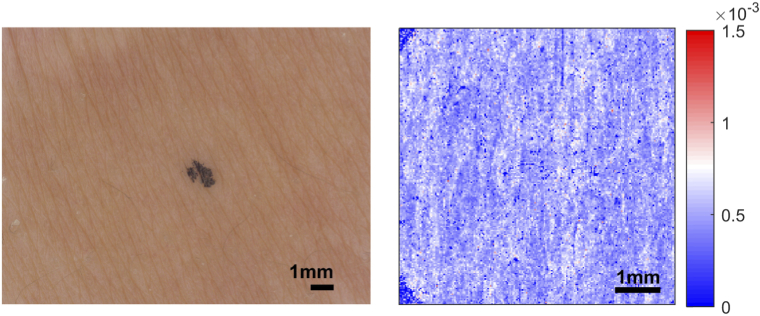
Example of healthy skin. Left: C-Cube image. Right: enface image of tissue birefringence. This sample was the corresponding healthy dataset to the striae alba sample in Fig. 8. N.B. the angular offset is not matched between the different imaging modalities.

In contrast, Fig. 9 shows an example of healthy skin. This sample is the corresponding healthy dataset to the striae alba sample in Fig. 8. Both the C-Cube and tissue birefringence images in Fig. 9 are more uniform in appearance.

Table 2 summarizes the number of samples categorized into each visual score category. The overall visual score is an average of 3 independent scorings, rounded to the nearest 0.5. Even though striae lesions were considered to be visually apparent during the screening, 6 of the striae-affected samples had a score of zero (no visible striae). This is because the scoring is judged from the C-Cube images and we sometimes find, particularly for the non-pigmented striae alba, that the striae-affected skin do not look like they have striae under the controlled lighting conditions of the C-Cube camera. C-Cube images with striae rubra are more likely to be given a higher score compared to images with striae alba. This is most likely due to a higher colour contrast between the striae and the surrounding healthy skin.

**Table 2.** Number of striae-affected samples in each C-Cube visual score category

Averaged score	Description	No. of samples
**0**	no visible striae	6
**0.5**		4
**1**	barely perceptible striae	32
**1.5**		8
**2**	visible striae	16
**2.5**		3
**3**	clearly visible striae with evidence of deep furrows	6

### 3.1 Statistical analysis

The result of the image processing steps described above yields a 200×200-pixel enface image and brightness histogram of the average tissue birefringence. A number of metrics were used to determine the best method of describing striae severity including: the arithmetic mean of the birefringence values, the median average and the arithmetic mean of the 75^th^ and 90^th^ percentiles. All analyses were carried out using MATLAB (2019a, MATLAB).

Using an unpaired two-sampled *t*-test, we aim to determine if the distribution of the means, defined in these 4 ways, are statistically different between striae-affected datasets (n=75) and the visually normal datasets (n=75). Table 3 shows the *p*-value of the tests for the four types of mean. The *p*-value is less than 0.05 for all of the metrics used. Thus, we can reject the null hypothesis at the 5% significance level and conclude that the SD datasets show significantly different tissue birefringence values compared to the visually normal datasets.

**Table 3.** Results of t-test for various tissue birefringence metrics

Metric	*p*-value of *t*-test	Mean and standard deviation of datasets	
		Striae	Visually normal
**Mean**	4×10^−6^	6.5 ± 1.7 ×10^−4^	5.4 ± 0.9 ×10^−4^
**Median**	3×10^−6^	6.1 ± 1.2 ×10^−4^	5.4 ± 0.9 ×10^−4^
**75^th^ percentile**	48×10^−6^	7.5 ± 1.9 ×10^−4^	6.3 ± 1.0 ×10^−4^
**90^th^ percentile**	3×10^−6^	9.2 ± 3.5 ×10^−4^	7.3 ± 1.0 ×10^−4^

The tests show that the tissue birefringence values show a greater standard deviation for the striae datasets compared to the visually normal datasets, indicating greater variation in tissue birefringence of striae skin compared to visually normal skin. Among the different metrics used, the 90^th^ percentile values show the greatest standard deviation for striae datasets. This suggests that it is the best candidate for differentiating striae severity based on the metrics considered here.

However, these differences are not large enough to place an unknown sample into either a striae or visually normal category with high accuracy. One reason for this may be because the visually normal samples were collected from a location that is adjacent to the striae samples. There is a possibility that the healthy samples also contain striae albeit with a lower severity. For a more conclusive verdict, more data is needed from a control group of healthy volunteers with no presentations of striae anywhere on the body. This would give us a benchmark for which to determine the healthy range of tissue birefringence measured using the PS-OCT.

**Fig. 10.**
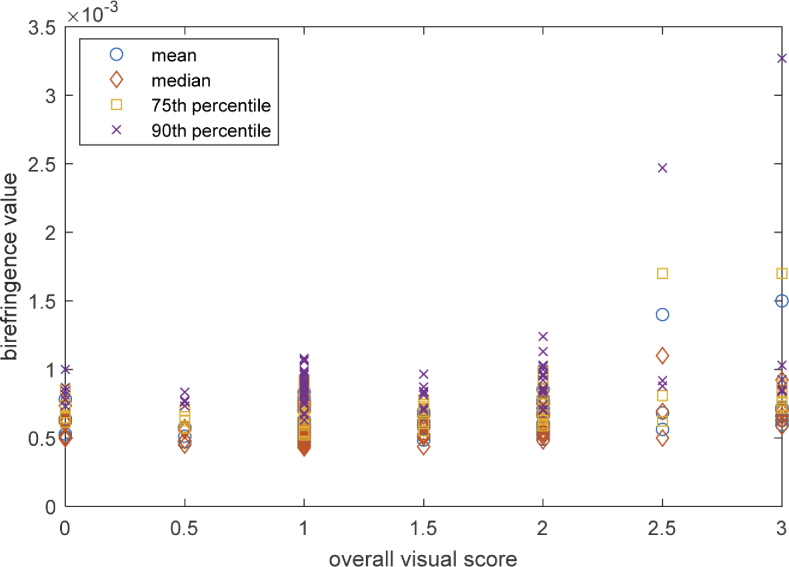
Graph comparing the correlation between each of the four tissue birefringence metrics (mean, median 75^th^ and 90^th^ percentile) against the corresponding C-Cube scores. These metrics were calculated for each tissue birefringence image (each open circle of a particular colour represents a given metric for a particular image) in order to determine whether a reliable birefringence-based metric can be used to represent striae severity. A linear regression line was calculated for each metric and the coefficient of determination was calculated. All show a weak correlation between the tissue birefringence scores and C-Cube scores, as indicated by Table 4.

These metrics were calculated for each dataset, plotted against the corresponding C-Cube score and the coefficient of determination (R^2^) was calculated. Figure 10 plots the different tissue birefringence metrics calculated for each enface image against the C-Cube visual scores. There is greater variation of tissue birefringence values in samples with higher visual scores compared to those with lower scores. A regression line was fitted to the plots. Table 4 shows the coefficient of determination (R^2^) for the metrics used. All have similar R^2^ values, indicating a weak correlation between the visual scores and the tissue birefringence. No one metric is markedly better than the other in terms of their correlation with the C-Cube scores. A reason for the low R^2^ value could be that the data is heavily skewed towards the lower visual scores; almost half of all striae samples have a visual score of 1. The weak correlation might suggest that PS-OCT is detecting complimentary information to visible-light dermoscopy.

**Table 4.** Coefficient of determination for different metrics used to represent striae severity

Metric	Gradient of linear regression	Coefficient of determination (R^2^)
**Mean**	7.1×10^−5^	0.11
**Median**	4.2×10^−5^	0.08
**75^th^ percentile**	8.4×10^−5^	0.12
**90^th^ percentile**	15.7×10^−5^	0.12

A higher tissue birefringence value is expected for SA compared to SR, as a key feature of SA is the presence of densely packed collagen fibers that run parallel to the surface of the skin [6]. However, as we only have 11 samples of SR compared to 60 samples for SA, it is not possible to determine whether this is true, with high statistical confidence. More samples for SR are needed. Furthermore, different patients may exhibit different tissue birefringence values due to differences in striae severity, genetics, etc. A larger sample size is needed to conclude whether tissue birefringence differs significantly between SR and SA.

## 4. Conclusions

To the best of our knowledge, this is the first exploratory study comparing visible-light dermoscopy with enface PS-OCT tissue birefringence imaging to assess the severity of striae distensae, in a clinically useful cohort size (150 measurement sites in 19 volunteers). Measuring the severity of striae would be useful, for example in assessing the effectiveness of treatment options and has, thus far, been done mostly by subjective visual scoring. The C-Cube camera captures images under fixed lighting conditions which are then given a visual score. We attempt in this paper to compare this method of scoring to quantified tissue birefringence metrics obtained using a PS-OCT system. It is hypothesized that the PS-OCT, with its ability to measure the sub-surface local birefringence of tissues, will provide score that is more reflective of the underlying physiology of striae.

Our data demonstrates that there is a statistically significant difference between visually normal skin and striae-affected skin. Using enface images of tissue birefringence, the striae samples are shown to have a higher mean, median, 75^th^ and 90^th^ percentile values in comparison to the visually normal samples. The 90^th^ percentile value seems to have a larger standard deviation among striae samples compared to the other metrics, suggesting that it is the best metric for differentiating SD severity. There seems to be a loose correlation between the tissue birefringence percentile scores and the C-Cube scores.

PS-OCT may thus show promise as a method for assessing striae severity, either alone or in combination with other modalities, but more studies are needed before it meets the requirements of a gold standard. Further potential investigations include collecting data on a control group of healthy volunteers and improving image processing techniques by increasing the 2D averaging pool size.

Recent developments in PS-OCT, such as Jones matrix PS-OCT systems [29], could give more insights into the spatial variation of local birefringence and optic axis that characterize SD. We are actively pursuing these new developments in our lab. A similar study investigating the local birefringence of human burn scars using Jones matrix OCT has successfully shown the efficacy of this technique [30]. Quantifying the spatial variation in Stokes vectors due to multiple scattering [31], modifying the scanning probe to include fiducial markers and the use of vascular masking techniques to aid in artefact removal [32] are also potentially useful avenues to explore.

As corticosteroid usage is a risk factor for SD [3], we are also developing PS-OCT in combination with structural and angiographic OCT to compare the effect of topical corticosteroids and new treatments for atopic dermatitis on the epidermis and dermis.
